# Do Relaxin Levels Impact Hip Injury Incidence in Women? A Scoping Review

**DOI:** 10.3389/fendo.2022.827512

**Published:** 2022-02-04

**Authors:** Emily A. Parker, Alex M. Meyer, Jessica E. Goetz, Michael C. Willey, Robert W. Westermann

**Affiliations:** ^1^ Department of Orthopedics and Rehabilitation, University of Iowa Hospitals and Clinics, Iowa City, IA, United States; ^2^ Orthopedic Biomechanics Laboratories, Department of Orthopedics and Rehabilitation, University of Iowa Hospitals and Clinics, Iowa City, IA, United States

**Keywords:** hip preservation, sex differences, female reproductive cycle, relaxin, sex-based, menstrual cycle hormones, hormonal contraceptives

## Abstract

**Purpose:**

The aim of this review is to assess the current evidence regarding the impact of relaxin on incidence of soft tissue hip injuries in women.

**Methods:**

A trained research librarian assisted with searches of PubMed, Embase, CINAHL, and SPORTDiscus, with a preset English language filter. The review was completed per the Joanna Briggs Institute (JBI) Manual for Evidence Synthesis methodology. Included studies required assessment of relaxin effects on musculoskeletal health, pelvic girdle stability, or hip joint structures in human subjects. Letters, texts, and opinion papers were excluded.

**Results:**

Our screen yielded 82 studies. Molecularly, relaxin activates matrix metalloproteinases (MMPs) including collagenases MMP-1/-13 and gelatinases MMP-2/-9 to loosen pelvic ligaments for parturition. However, relaxin receptors have also been detected in female periarticular tissues, such as the anterior cruciate ligament, which tears significantly more often during the menstrual cycle peak of relaxin. Recently, high concentrations of relaxin-activated MMP-9 receptors have been found on the acetabular labrum; their expression upregulated by estrogen.

**Conclusions:**

Menstrual cycle peaks of relaxin activate MMPs, which locally degrade collagen and gelatine. Women have relaxin receptors in multiple joints including the hip and knee, and increased relaxin correlates with increased musculoskeletal injuries. Relaxin has paracrine effects in the female pelvis on ligaments adjacent to hip structures, such as acetabular labral cells which express high levels of relaxin-targeted MMPs. Therefore, it is imperative to investigate the effect of relaxin on the hip to determine if increased levels of relaxin are associated with an increased risk of acetabular labral tears.

## Introduction

Female athletes still face sex-based disparity in sports-related injury with significantly lower likelihood of a healthy career. One in five female collegiate athletes will suffer an anterior cruciate ligament (ACL) injury in college (1–3). ACL ruptures are a more evident, more thoroughly researched female-predominant injury (4–6); females in this age group are also more likely to undergo surgical treatment for athletic hip injuries. Multiple factors contribute to the female predominance of ACL injuries, including neuromuscular discrepancies and fluctuating levels of reproductive hormones (7). Hormonal research has recently identified a promising target: relaxin (1, 7–10). This has not been investigated in relation to young athletic hip conditions.

Relaxin cycles with other menstrual hormones and weakens collagen in target tissues such as the pubic symphysis (1, 9–14). While necessary for parturition, this can be detrimental outside of the reproductive system (15–17). Multiple studies correlate relaxin peaks with female ACL tears (1, 9). However, despite large-scale relaxin synthesis in the pelvis, and a known paracrine activity profile, there is a paucity of literature assessing if relaxin levels correlate with another female-predominant lower extremity injury impacting athletes such as acetabular labral tears (18–21). If relaxin does significantly contribute to the elevated rates of both knee and hip injuries in women, further research into preventive strategies is critical.

The absence of literature regarding relaxin vs. hip injuries necessitated a scoping review to appraise available information on factors related to relaxin versus hip injury, and to identify conceptual gaps (22). This review assessed relevant literature on nanoscale, microscale, and macroscale actions of relaxin. The objective of this scoping review is to show that it is scientifically logical and medically important to further explore the potential correlation between relaxin levels and female hip injuries.

## Methods

A preliminary search of MEDLINE, the Cochrane Database of Systematic Reviews and JBI Evidence Synthesis (Joanna Briggs Institute, Adelaide, Australia) was conducted and no current or underway systematic reviews or scoping reviews on the topic were identified. The scoping review was conducted in accordance with the JBI methodology for scoping reviews (JBI Manual for Evidence Synthesis) and the Preferred Reporting Items for Systematic reviews and Meta-Analyses for Scoping Reviews (PRISMA-ScR) Checklist (**Supplementary Material: Appendix 1**).

### Types of Sources

This scoping review considered all traditional types of papers, with the exclusion of commentaries, editorials, and opinion papers.

### Search Strategy

The search strategy was developed by the authors with the assistance of a Health Sciences librarian specializing in development of database queries. The search strategy aimed to locate both published and unpublished studies (“gray literature”). An initial limited search of MEDLINE and EMBASE was undertaken to identify articles on the topic. The index terms used to describe the articles were used to develop the full search strategy, along with text words derived from the titles and abstracts of relevant articles.

The initial search strategy was developed for MEDLINE. The first search concept focused on female hormonal variations, with MeSH terms such as estrogens, progesterone, relaxin, and contraceptives. Text words and phrases included “cyclic hormonal variation” and “female athlete hormonal variation”. The second search concept focused on non-arthritic hip pain, with MeSH terms such as hip dysplasia and femoroacetabular impingement. Text words and phrases included “acetabular labral tear” and “pelvic floor disorder”. The full search strategy for MEDLINE can be found in the Appendix (**Supplementary Material: Appendix 2**).

The search strategy was then adapted for EMBASE and CINAHL. Full search strategies for these databases are available upon request. The reference list of all included sources of evidence was screened for additional source materials *via* SCOPUS. A filter for English language studies was used. During the screening process, the decision was made to exclude animal-only studies.

### Source of Evidence Selection and Data Extraction

All identified citations were uploaded to EndNote (EndNote X9.2, Clarivate Analytics, PA, USA) and duplicates removed by a combination of software screening and manual review. Titles and abstracts were then screened by two authors independently against the inclusion criteria (**Table 1**). A full-text assessment was then performed to identify final inclusions. We elected to exclude animal-only studies, and include studies of pregnant women only for the pelvis and hip subsections. Any disagreements during the selection process were resolved by the senior authors.

**Table 1:** Scoping review screening, inclusion and exclusion criteria.

Inclusion	Exclusion
Adults and children Level I-V, unpublished (“gray”) literature Systematic Reviews/Meta-Analyses All publication dates Mixed studies with animal and human subjects • Ex) Molecular analysis of porcine and human ligaments Human cadaver studies Must address a scoping review question: • Relaxin and musculoskeletal health • Relaxin and pelvic girdle stability • Relaxin and hip structures	Non-English Animal only Commentary, editorial, letters, opinion statements, technical descriptions Studies addressing a key scoping review area (musculoskeletal health, pelvic girdle stability, and/or hip structures) which address relaxin in a manner with negligible extractable information • Ex) Analysis of hormone levels versus pelvic instability which only mentions relaxin briefly (“relaxin may also play a role in pelvic instability”)

Data was extracted by two authors, independently, with oversight from the senior authors. The data included subject demographics, concept/context, study methods, and key findings relevant to the present review questions. The data extraction approach was modified and revised as necessary. The data was documented in one or more analytical categories of relaxin effect level: cellular/molecular, systemic-musculoskeletal, pelvic structure-specific, and hip-specific.

Per scoping review protocol, critical appraisal of individual sources was not completed. The current review will not individually discuss all included studies. Discussion will address studies with novel information and/or critical concepts, which will be recorded in subject-specific tables (**Tables 2**–**4**). However, all included references are listed in **Supplementary Material: Appendix 3**.

**Table 2:** Cellular/molecular effects of relaxin- relevant literature findings.

Subcategory	Author, Year	Findings
Basic Properties of Relaxin	Goldsmith et al. (23)	Relaxin (RLX^*^) is a peptide hormone in the insulin-like growth factor (IGF^†^) family Men and women have similar serum levels (400-500 pg/mL and 360-495 pg/mL), with luteal phase peaks in women Oral contraceptives decrease relaxin below a detectable serum level The major gene for relaxin in humans is H2; H2 relaxin binds relaxin family peptide receptor 1 and 2 The corpus luteum produces most relaxin, but synthesis occurs in the endometrium, placenta, breast tissue, and prostate Relaxin is biologically and immunologically active during pregnancy The capacity of relaxin to act locally means that serum levels do not always reflect activity
	Grossman et al. (24)	
	Lubahn et al. (25)	
	MacLennan et al. (26)	
	Powell et al. (27)	
	Wolf et al. (17)	
	Wolf et al. (28)	
Properties of Relaxin Receptors	Bryant-Greenwood et al. (29)	In humans, relaxin family peptide receptor-1 (RXFP^‡^) is most common, and has the highest affinity for H2 relaxin Relaxin binds receptors in a time-, temperature-, and pH-dependent manner RXFP^‡^ expression is primed by estrogen/progesterone in chondroblasts, fibrochondroblasts, myofibroblasts, and ligaments Estrogen-primed receptors can show maximum response at RLX^*^ levels 10-100 times lower than normal Estrogen, progesterone, and relaxin receptors modulate MMP^§^ transcription and post-translational modification Radioreceptor location detection is a sensitive indicator of the physiological roles of RLX^*^ Relaxin receptors are detectable in anterior cruciate ligament (ACL^¶^) remnants of female, but not male, surgical patients RLX^*^ binding was uniform, saturable, and specific to the synovial lining, stromal fibroblasts, and intima. Relaxin receptors have been detected in the carpometacarpal joint of the thumb (1^st^ CMC^#^) in arthroplasty patients The synovial lining, dorsoradial ligament, volar oblique ligament, and articular cartilage cells had receptors Concentration of RLX^*^ receptors was significantly higher in women compared to men Relaxin receptors have been detected in the temporomandibular joint (TMJ^**^), on fibrochondrocytes and ligaments
	Dragoo et al. (10)	
	Kapila et al. (30)	
	Kleine et al. (31)	
	MacLennan et al. (26)	
	Powell et al. (27)	
Functional, Physiologic Properties of Relaxin	Ando et al. (32)	Relaxin controls extracellular matrix (ECM^††^) turnover by stimulating collagen degradation, and suppressing synthesis Relaxin upregulates MMP^¥^ production, specifically collagenases (MMP-1/-13) and gelatinases (MMP^§^-2/-9) Active collagenases cleave tropocollagen, making it susceptible to subsequent denaturation by gelatinases The density and organization of collagen bundles, and total local collagen content decrease MMP^§^s induced by relaxin degrade collagen at a nanoscale level, and macro-level effects are not always appreciable Relaxin has dose-dependent and differential functioning; its effects depend on location and presence of other hormones There is a significant correlation between peak serum relaxin and peak serum progesterone levels Intracellular relaxin activates MAPK^‡‡^ and PI3K^§§^, increasing cAMP^¶¶^ and triggering vasodilation *via* MMP^§^-2/-9 Estrogen, progesterone, and relaxin binding synovial receptors upregulates inflammatory MMP^§^s, increasing OA^##^ risk Relaxin upregulates production of collagenases and gelatinases in ligaments and fibrocartilage During parturition, relaxin binding pubic ligaments dissociates collagen, increases water uptake, and decreases viscosity
	Dragoo et al. (10)	
	Galey et al. (33)	
	Goldsmith et al. (23)	
	Grossman et al. (24)	
	Nose-Ogura et al. (13)	
	Powell et al. (27)	

^*^RLX, relaxin.

^†^IGF, insulin-like growth factor.

^‡^RXFP1 or 2, relaxin family peptide receptor 1 or 2.

^§^MMP, matrix metalloproteinase.

^¶^ACL, anterior cruciate ligament.

^#^1st CMC, first/thumb carpometacarpal joint.

^**^TMJ,temporomandibular joint.

^††^ECM, extra-cellular matrix.

^‡‡^MAPK, mitogen-activated phosphate kinase.

^§§^PI3K, phosphoinositide-3-kinase.

^¶¶^cAMP, cyclic adenosine monophosphate.

^##^OA, osteoarthritis.

**Table 3:** Musculoskeletal effects of relaxin- relevant literature findings.

Subcategory	Author, Year	Findings
Relaxin and Tendons, Ligaments of the Leg	Arnold et al. (8)	4-year careers of 128 Division 1 collegiate female athletes in sports with the highest ACL^‡^ tear risk—basketball, lacrosse, field hockey, and soccer— tested SRC^#^ during mid-luteal phase, CD^**^ 21-24 Cumulative career incidence of ACL^‡^ tears was 21.9%; associated average SRC^#^ was higher (6.0 ± 8.1 vs. 1.8 ± 3.4, p<0.013) Subgroup: 46/128 athletes with detectable SRC^#^- ACL^‡^ tear incidence was 30.4% (14/46) with associated average SRC^#^ 12.1 ± 7.7 (vs. 5.7 ± 3.6, p<0.002) Trial of ACL^‡^ injury risk screening at SRC^#^ 6.0 pg/mL: Screen was 71% sensitive, 69% specific; PPV^‡‡^ 52%, NPV^††^ 88% Conclusion: Elite female athletes with SRC^#^>6.0 pg/mL had 4 times more ACL^‡^ tears (RR^***^ 4.4, χ^2^ p=0.003, ROC^§§^ 0.002) A separate analysis of the same 128 D1 female athletes assessed SRC^#^ vs. menstrual cycle status With OCP^##^ use: SRC^#^ 1.41 (vs. 3.08, p<0.002); significant lower SPC^¶¶^ was also seen (2.8 vs. 6.99, p<0.0002) Without OCP^##^ use: No significant SRC^#^ difference in eumenorrheic vs. amenorrheic vs. oligomenorrheic athletes Sex-specific neuromuscular differences account for some disparity in ACL^‡^ tear rates, but hormonal differences are also involved Current biomechanics training has some success, role of fatigue in competition unknown ACL^‡^ estrogen/progesterone/relaxin/testosterone receptors; due to the complexity of hormone signaling, single-timepoint analyses are not reliable 165,748 females ACL^‡^ reconstruction patients assessed for use of OCPs^##^: OR^***^ for ACL^‡^ tear on OCPs^##^ was 0.82 Most significant in 15-19 yo age group with OR^***^ 0.37, a 63% risk reduction, NNT^†††^ of 6 Eumenorrheic females, no OCPs^##^: Luteal phase SRC^#^ peaks correlated with laxity of patellar tendon, no change in gastrocnemius
	Brophy et al. (7)	
	Clifton et al. (34)	
	Dragoo et al. (1)	
	Dragoo et al. (9)	
	Pearson et al. (35)	
Relaxin and the Thumb CMC^§^ Joint	Komatsu et al. (36)	CMC^§^ arthroplasty patients with elevated SRC^#^ expressed increased RXFP1^‡^ in nearby ligaments RXFP1^‡^ upregulates MMP1➔ increases joint laxity, abnormal loading CMC^§^ subluxation risk positively correlates with detectable SRC^#^ Effects of relaxin should be considered during CMC^§^ ligament repairs in women of childbearing age
	Wolf et al. (15)	
	Wolf et al. (4)	
Relaxin and the Jaw, Mouth	Deniz et al. (14)	TMJD^¶^ patients with OA^‡‡‡^ and joint effusion had higher synovial fluid relaxin levels vs. TMJD^¶^ patients with OA^‡‡‡^ alone Weekly gingival relaxin injections did not impact tooth movement during adjustive treatment
	Deniz et al. (37)	
	McGorray et al. (38)	
Relaxin and the Shoulder	Owens et al. (39)	Military cadets with an episode of acute shoulder instability (47M:6F); were compared to age/sex/height/weight matched controls Those with instability had higher SRC^#^ (3.69 vs. 2.20, p=0.02) For every 1 pg/mL increase in SRC^#^ at baseline, cadets were 2.18 times more likely to have an episode (95% CI 1.01-4.76)

^*^RLX, Relaxin.

^†^RXFP1, RXFP2, Relaxin family peptide receptor 1, 2.

^‡^ACL(R), Anterior cruciate ligament (repair).

^§^1^st^ CMC, First/thumb carpometacarpal joint.

^¶^TMJ(D), Temporomandibular joint disorder.

^#^SRC, Serum relaxin concentration.

^**^CD, [Menstrual] cycle day.

^††^NPV, Negative predictive value.

^‡‡^PPV, Positive predictive value.

^§§^ROC, Receiver operator curve.

^¶¶^SPC, Serum progesterone concentration.

^##^OCP, Oral contraceptive.

^***^OR/RR, Odds ratio, risk ratio.

^†††^NNT, Number needed to treat.

^‡‡‡^OA, Osteoarthritis.

**Table 4:** Pelvic and hip joint related effects of relaxin- relevant literature findings.

Subcategory	Author, Year	Findings
Relaxin and the Pubic Symphysis	MacLennan et al. (40)	135 women with pubic symphysis disorder (SPD^*^) had SRC^†^ above the 95% percentile for an average female population Infants with DDH^‡^ born to mothers with PS^§^ instability will also have PS^§^ instability on exam Pubic symphysis: Fibrocartilaginous joint, supported by symphyseal ligaments, arcuate ligaments between inferior pubic rami, posterior sacral ligaments, and iliolumbar ligaments—outside of pregnancy, takes 2600 lbs of force to separate
	MacLennan et al. (41)	
	Schuster et al. (21)	
Relaxin and Uterine Ligaments	Kieserman-Schmokler et al. (42)	Uterine prolapse patients have significantly higher SRC^†^, R2 *in utero*sacral ligaments—modulated by oxytocin and relaxin Female infants with DDH^‡^ have an 11.2 times higher inguinal hernia risk in their first 3 months of life; have surgery earlier (1 mo vs. 10 mo) Of all operations on female infants <3 mo of age for inguinal hernias, 25% have DDH^‡^
	Reisenauer et al. (43)	
	Schott et al. (44)	
	Uden et al. (45)	
Relaxin Peripartum	Bookhout et al. (46)	Pregnant women with pelvic pain and pelvic joint instability (PPPJI^¶^) were diagnosed earlier if multiparous, prior OCP^#^ use Severe PPPJI^¶^ symptoms in the 3^rd^ trimester correlated with higher SRC^†^ Infants of PPPJI^¶^ mothers tended to be post-term, higher weight, and female; 25 in 1000 had DDH^‡^ 25% of women will have disabling musculoskeletal pain of the pelvis/low back at some point during pregnancy Primigravid women had a significant positive correlation between SRC^†^ and pelvic/back pain, stratified at 36 weeks SRC^†^<420: 20% had lumbosacral and PS^§^ pain SRC^†^ 420-890: 45% had lumbosacral and PS^§^ pain SRC^†^>890: 55% had lumbosacral and PS^§^ pain, 10% had greater trochanteric pain SPD^*^ occurs in 1/36 pregnancies; worse PS^§^ pain correlates with more PS^§^ separation; acute PS^§^ disruption risk is 1:300-1000
	Kristiansson et al. (47)	
	Ritchie et al. (48)	
	Saugstead et al. (49)	
Relaxin Postpartum	Borg-Stein et al. (50)	Women with higher SRC^†^ during pregnancy take significantly longer to recover Postpartum relaxin does not return to baseline until 4-12 weeks; injury risk remains elevated; leg and foot pain twice as likely
	Leadbetter et al. (51)	
Relaxin and Maternal Factors Impacting DDH^‡^	Andren et al. (18)	If the uterine wall does not put normal pressure on the femoral head/acetabular socket interface, DDH^‡^ occurs Two studies found that maternal SPD^*^ increased infant DDH^‡^ risk five-fold; one postulated a genetic susceptibility to relaxin A majority of DDH^‡^ infants had primigravid mothers; risk is also increased in twin births, (monozygotic>dizygotic) A sibling with DDH^‡^ increases risk 4.3-14%, while a parent with DDH^‡^ increases risk 1.6-2.3% No association between cord blood SRC^†^ and DDH^‡^ diagnosis; in two studies DDH^‡^ infant cord blood SRC^†^ was mildly lower Lower maternal relaxin was theorized to decrease laxity of the birth canal
	Bracken et al. (52)	
	Forst et al. (53)	
	MacLennan et al. (19)	
	Roof et al. (54)	
Relaxin and Fetal Factors Impacting DDH^‡^	Andren et al. (18)	DDH^‡^ risk factors, research supported: breech, family history, firstborn, oligohydramnios, high birth weight, postmaturity However, 73-90% of infants with DDH^‡^ have no identifiable risk factors other than female sex Female fetuses are more responsive to maternal hormones, but normally metabolizes and excretes them (hepatic metabolism) DDH^‡^ could reflect decreased ability to metabolize/increased sensitivity to hormones Neonates with DDH^‡^ and abnormal estrogen excretion tend to have PS^§^ instability Could be an inborn, possibly hereditary error of estrogen metabolism DDH^‡^ risk, female infants: 19/1000 baseline, 44/1000 with family history, 120/1000 with breech birth; 25/1000 in PPPJI^¶^ moms 80% of DDH^‡^ cases are bilateral, unilateral cases are 4 times more likely to be left sided due to intrauterine positioning
	Bracken et al. (52)	
	Forst et al. (53)	
	Morey et al. (20)	
	Rhodes et al. (55)	
	Roof et al. (54)	
	Schuster et al. (21)	
	Uden et al. (45)	
Relaxin and Neonatal Findings	Andren et al. (18)	In a study of 90 DDH^‡^ neonates, a majority of infants had concurrent pelvic instability on exam Abnormalities on neonatal hip ultrasound are significantly more likely to spontaneously resolve in males Female neonates with DDH^‡^ have an 11.2 times greater risk of developing an inguinal hernia during their first 3 months of life
	Bracken et al. (52)	
	Uden et al. 45)	

^*^SPD, symphysis pubis dysfunction/pubic symphysis dysfunction.

^†^SRC, serum relaxin concentration.

^‡^DDH, developmental dysplasia of the hip.

^§^PS, pubic symphysis.

^¶^PPPJI, pelvic pain and pelvic joint instability.

^#^OCP, Oral contraceptive.

## Data Analysis Results

Of the initial library screened, 82 articles were included for scoping review (1, 4, 5, 7–21, 23–86). There was overlap between analysis categories for numerous papers; most prevalent with the subjects are of cellular/molecular and systemic-musculoskeletal effects. The effects of relaxin were discussed at the cellular/molecular level by 24 studies (10, 13, 16, 17, 23–33, 58, 62, 68, 71, 76, 77, 80, 83, 84), and at the systemic-musculoskeletal level by 25 studies (1, 4, 7–9, 11, 12, 14, 15, 34–39, 57, 60, 61, 63, 64, 67, 75, 81, 82, 85). The pelvic structure-specific effects of relaxin were discussed by 17 studies (21, 40–51, 59, 73, 74, 86), and 9 studies focused on hip joint-specific relaxin effects (18–21, 45, 52–55).

## Data Presentation and Discussion

The present review identified conceptual gaps regarding the intersection of relaxin levels and female hip injuries, and appraised available information on factors related to the topic, with review of 82 studies. Literature data was documented in conceptual categories of relaxin effects. Important/main concepts from the data assessed for each category are reported in the following sub-sections.

## Cellular/Molecular Effects of Relaxin

### Basic Properties of Relaxin

**Figure 1:**
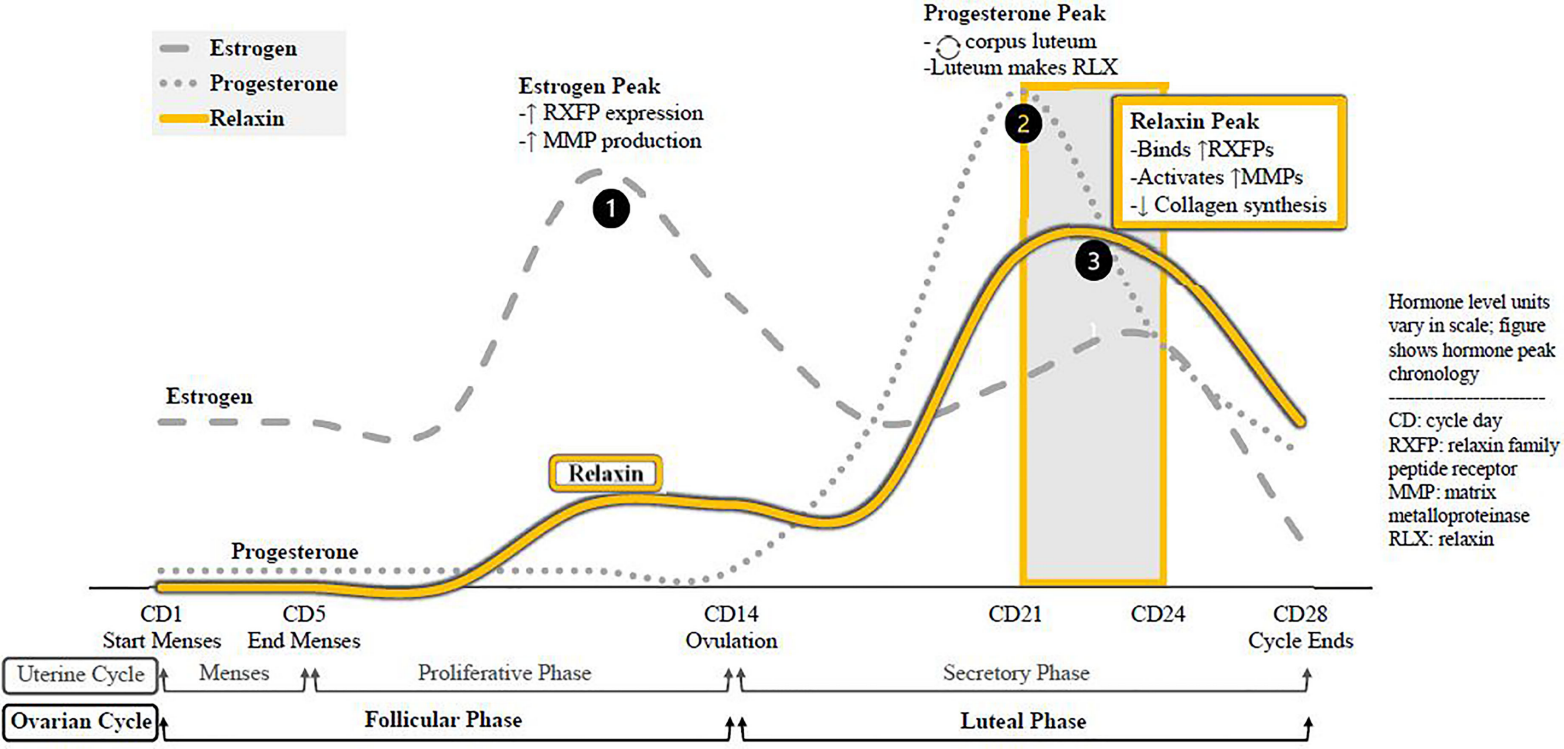
Menstrual cycle hormone peaks, molecular effects. The sequence of hormone peaks for ovulatory cycles. Estrogen levels peak first, increasing expression of relaxin receptors in the body and increasing global synthesis of MMPs. The drop in estrogen triggers ovulation, and the remains of that ovarian follicle from the corpus luteum. As a temporary endocrine body, the corpus luteum secretes progesterone to prepare the endometrium for pregnancy and to sustain itself. It also synthesizes and relaxin, which binds receptors and activates. MMPs recently upregulated by estrogen while suppressing de novo collagen synthesis. Relaxin is active during the luteal phase, chiefly CD21-24.

Relaxin is a peptide hormone present in both sexes, with known paracrine, autocrine, and endocrine actions. The corpus luteum synthesizes the bulk of relaxin, but the endometrium, placenta, breast tissue, and prostate have also been detected as synthesis sites. Average serum relaxin concentration (SRC) is similar between non-pregnant women and men; although women’s levels peak at menstrual cycle day 21-24. As relaxin often acts in a paracrine fashion, SRC does not consistently reflect the extent of hormone activity (**Table 2**). The menstrual cycle peaks and corresponding molecular changes in the body for the three essential menstrual hormones—estrogen, progesterone, and relaxin—are depicted in **Figure 1**.

### Properties of Relaxin Receptors

The location of relaxin family peptide receptors (RXFPs) is a sensitive indicator of physiological roles. Thus, the curiosity regarding sex-specific musculoskeletal roles of relaxin, as RXFPs are uniformly present—only in women—in the synovial lining of ACL remnants, and present in high concentrations in women undergoing first carpometacarpal arthroplasty. Additionally, RXFP expression is primed by estrogen and progesterone (**Table 2**).

### General Functional and Physiologic Properties of Relaxin

**Figure 2:**
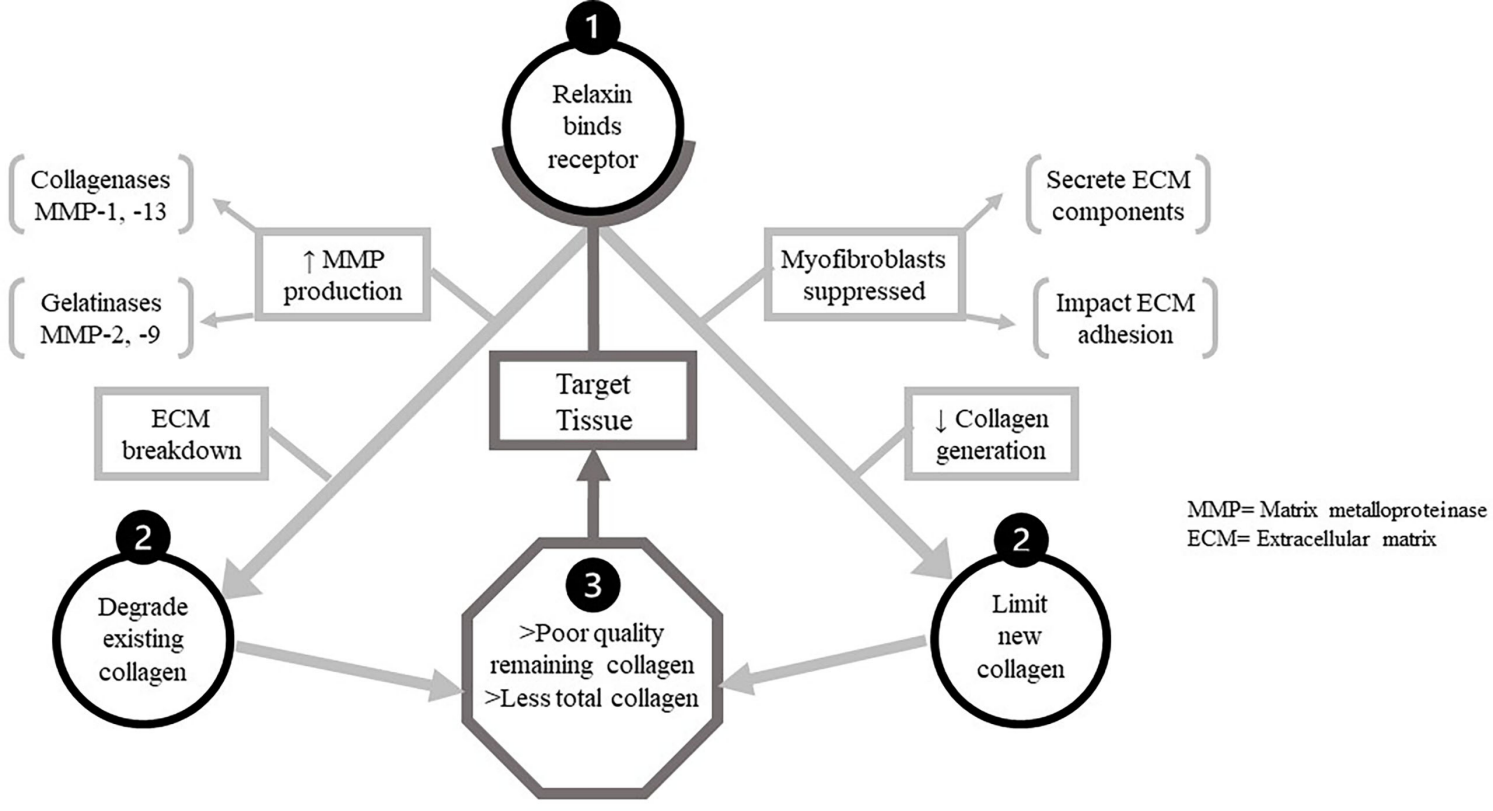
The dual functions of relaxin in target tissues. The two main mechanistic pathways by which relaxin decreases tissue quality and quantity of collagen after binding to its receptor. Upon binding relaxin receptors in target tissue, two processes impacting collagen proceed concurrently. Above, left-relaxin increases production of all MMPs, but particularly the collagenases and gelatinases capable of digesting ECM components. Thus one mechanistic pathway detrimental to target tissue collagen is the degradation of existing collagen by these. MMPs. Above, right-relaxin also suppresses function of/differentiation into myofibroblasts. These cell secrete multiple ECM components and modulate ECM cross-linkage, allowing relaxin to impair de novo collagen synthesis is target tissue.

Relaxin is a controller of ECM turnover, upregulating MMP-1/-13 (collagenases) and MMP-2/-9 (gelatinases) to degrade existing collagen while suppressing synthesis of new collagen. Poor collagen quality in the target tissues, along with a lesser amount of proximate total collagen, are the result of these complementary actions of relaxin; illustrated in **Figure 2**.

## Key Reference One: Roles of Matrix Metalloproteinases

Relaxin modulates many of its effects *via* MMPs, whose roles were detailed in a 2011 review article by Klein et al. (31) MMP-1 uniquely degrades triple-helical (fibrillar) collagen, leaving it susceptible to gelatinases. Dysregulation causes insufficient or excessive tissue regeneration. MMP-13, activated by MMP-3, cleaves type II collagen during bone remodeling. Dysfunction has been linked to cartilage destruction in osteoarthritis and rheumatoid arthritis. MMP-2 degrades gelatine (denatured collagen) and is constitutively expressed. It has roles in angiogenesis and basement membrane structure. MMP-9 also degrades gelatine and is highly inducible by other MMPs. It signals immune cells and is crucial for endometrial tissue remodeling during the menstrual cycle. MMP-3 degrades numerous ECM proteins and is involved in activation of gelatinases and MMP-13 (31).

## Systemic-Musculoskeletal Effects of Relaxin

### Anterior Cruciate Ligament

The bulk of literature directly addressing relaxin and sex-specific disparities in musculoskeletal health focuses on incidence of ACL rupture. It has been cited that a combination of neuromuscular/biomechanical differences and sex-specific hormonal variations are responsible for the high rate of female ACL injuries. Current injury prevention programs to correct biomechanics have had some success decreasing ACL rupture rates, but a systematic review of prevention program studies concluded that neuromuscular differences alone could not account for the disparity. The female ACL contains receptors for estrogen, progesterone, relaxin, and testosterone, and coupled with the complexity of the hormonal cycle, the authors also noted that single-timepoint hormonal analyses were not depictive (**Table 4**). Additionally, two studies found that in males, their naturally higher testosterone levels have a protective effect on the ACL, by downregulating collagenolytic activity (87, 88).

ACL tear incidence in elite female athletes is as high as 21.9%, as demonstrated in a study which included nearly 130 Division 1 (D1) female collegiate athletes during their 4-year careers in sports with a high risk of ACL injury: basketball, lacrosse, field hockey, and soccer. The athletes underwent SRC testing during the mid-luteal phase of their menstrual cycle (day 21-24), which showed significantly higher average SRC among athletes who had, or would, suffer ACL tears (6.0 ± 8.1 vs. 1.8 ± 3.4, p<0.013) (**Table 3**).

Given that SRCs are not always detectable, a subgroup of the 46 athletes with detectable mid-luteal SRC were analyzed. The ACL tear incidence of this subgroup was 30.4% (14/46). Average SRC of injured athletes was 12.1 ± 7.7, vs. 5.7 ± 3.6 in those without injury (p<0.002). Among this group, researchers trialed an SRC of 6.0 pg/mL as the cutoff level for ACL injury risk screening. This screen proved to be 71% sensitive and 69% specific, with a PPV of 52% and an NPV of 88%. Researchers concluded that elite female athletes with SRC>6.0 pg/mL had a 4-fold increased risk of an ACL tear during their career (RR 4.4, χ^2^ p=0.003, ROC =0.002) (**Table 3**).

This group of D1 athletes was also surveyed about oral contraceptive pill (OCP) use, to assess potential effects on SRC and ACL tears. It was found that athletes taking OCPs had significantly lower average SRC (1.41 vs 3.08, p<0.002) and average serum progesterone concentration (SPC) (2.8 vs. 6.99, p<0.0002). Interestingly, SRC was not significantly different between eumenorrheic, amenorrheic, and oligomenorrheic athletes. While this cohort study showed that OCPs significantly reduce SRC, a database review of nearly 170,000 female ACL reconstructions showed that OCP also reduced the likelihood of an ACL tear (0.82). This was most significant among 15-19 year-olds, with likelihood of an ACL tear decreasing by 63% (OR 0.37). Analyzing OCPs as a preventive treatment for ACL tears in this age group yielded an NNT of 6 adolescent athletes (**Table 3**).

### First CMC Joint (Trapeziometacarpal Joint)

First CMC arthroplasty is a common procedure among women. Studies of first CMC instability and degeneration found the risk of subluxation to be positively correlated with detectable SRC. Immunohistochemical studies of tissue from arthroplasty patients with elevated average SRC showed increased expression of the relaxin receptor RXFP1 in nearby ligaments subsequently increasing collagen degradation leading to increased CMC laxity and abnormal weight loading (**Table 3**).

### Other Musculoskeletal Tissues

Patients with temporomandibular joint disorder (TMJD), another female-predominant condition, showed higher average synovial fluid relaxin levels in those with TMJ OA plus joint effusion, rather than OA alone. Shoulder instability, an issue common in young female athletes playing overhead sports, was assessed in a cohort study of 53 military cadets (47 male, 6 female) with episodes of acute shoulder instability. Compared to controls matched for age, sex, height, and weight, these 53 cadets had significantly higher SRC (3.69 vs. 2.20, p=0.02). Risk of an acute instability episode vs. SRC had a dose-dependent correlation; every 1 pg/mL increase in baseline SRC making acute instability 2.18 times more likely (95% CI 1.01-4.76) (**Table 3**).

## Pelvic Structure-Specific Effects of Relaxin

### Pubic Symphysis

The fibrocartilaginous pubic symphysis joint is stabilized by a combination of symphyseal ligaments, arcuate ligaments between inferior pubic rami, posterior sacral ligaments, and iliolumbar ligaments. Normally, this joint takes 2600 lbs of force to separate; but during late pregnancy and parturition, relaxin triggers enough collagen degradation for separation to occur at much lower forces. In women with the musculoskeletal/genitourinary symptoms of symphysis pubis dysfunction (SPD) their average SRC was above the 95th percentile for average SRC distribution among age and sex matched controls (**Table 4**).

### Uterine/Pelvic Ligaments

Relaxin and oxytocin modulate the integrity of uterosacral ligaments, and uterine prolapse patients had significantly higher SRC versus controls, as well as significantly increased RL-2 in ligament samples from prolapse patients vs. control patients undergoing gynecologic surgery. Even prior to puberty relaxin impacts pelvic soft tissues. During the first three months of life, female infants with developmental hip dysplasia (DDH), theorized to have an associated with excess relaxin, have an 11.2 times greater risk of developing an inguinal hernia, and tend to require earlier surgical repairs (1 mo vs. 10 mo). Of all female infants less than 3 months old who require operative intervention for inguinal hernias, 25% have DDH (**Table 4**).

### Peripartum Pubic Symphysis, Lumbosacral Joints

A study of pregnant women with pelvic pain and pelvic joint instability (PPPJI) found earlier onset and increased severity in patients with a history of OCP use (exogenously lowered relaxin) and for multiparous women (prior pelvic ligament degradation). Those with severe 3^rd^ trimester symptoms had significantly higher SRC. Their infants tended to be post-term, higher weight, and female; and approximately 25/1000 had DDH. A study of primigravid women at 36 weeks recorded pain incidence and location stratified by mean SRC. Of women with low SRC (<420 pg/mL), 20% had lumbosacral and PS pain. In women with moderate SRC (420-890 pg/mL), 45% had this pain, and which bothered 55% of women with high SRC (>890 pg/mL); 10% of whom also had greater trochanteric pain. Research found that 25% of pregnant women will at some point experience disabling musculoskeletal pain of spine and/or pelvis.

### Postpartum Pelvic Recovery

Women with higher peripartum SRC took longer to recover from childbirth. Relaxin does not return to baseline SRC until 4-12 weeks postpartum, and women are 2 times more likely to have leg and foot pain secondary to pelvic instability during this period (**Table 4**).

## Hip-Specific Effects of Relaxin

### Fetal Factors Related to DDH

DDH has well-defined risk factors, including breech delivery, female sex, family history, firstborn status, high birth weight, and post-maturity. However, 73-90% of infants with DDH have no identifiable risk factor other than female sex; generating the hypothesis that female fetuses are more susceptible to maternal hormones. In normal physiological states, the fetal liver will metabolize these hormones. This is one potential “problem point” leading to DDH; if the fetus has decreased metabolic abilities or increased hormonal sensitivity. Given that neonates with abnormal estrogen excretion and DDH commonly have PS instability on exam, an inborn and possibly hereditary error of estrogen metabolism could be a contributing factor (**Table 4**).

### Maternal Factors Related to DDH

*In utero*, if the maternal uterine wall does not put normal pressure on the fetal femoral head/acetabular socket interface, DDH results. If pregnant women have persistent pelvic/PS pain, their infants have a 5 times increased risk of DDH. The risk of DDH for female infants at baseline was listed at 19:1000 (“normal” risk of DDH varied by source, but generally ranged between 14-20:1000). Per SPD research, women with more pelvic pain during pregnancy likely have significantly increased SRC, and as previously discussed studies of mothers with SSPJI, symptomatic SPD in pregnancy increases DDH risk to approximately 25:1000 (**Table 4**).

Primigravid mothers and those with multiple gestation pregnancies, monozygotic more than dizygotic, have an increased risk of neonate DDH. No significant association has been established between cord blood SRC and incidence of DDH; two studies in this review noted insignificantly lower SRC in cord blood of infants with SRC. It was not known if this could be secondary to inadequate relaxation of the birth canal; or if cord blood SRC was not useful to analyze in this situation given the known local effects of relaxin (**Table 4**).

### Physical Findings Indicating Excess Relaxin in Neonates with DDH

A study of 90 neonates with DDH detected concurrent pelvic instability on physical exam in a majority of neonates. Abnormalities detected on neonatal hip ultrasound were significantly more likely to spontaneously resolve in male infants. Additionally, further supporting the theory of pelvic effects of relaxin, female neonates with DDH have an 11.2 times greater risk of developing an inguinal hernia during the first three months of life (**Table 4**).

## Key Reference Two: Metabolism of Cells of the Acetabular Labrum

A critical scientific component linking known actions of relaxin to a potential impact on hip health is research by Dhollander et al (89) exploring the cellular metabolism of human acetabular labral cells. Analysis was performed on articular and capsular side labral cells, articular chondrocytes, and meniscal cells (fibrochondrocytes). Labral cells had a number of properties similar to meniscal fibrochondrocytes, such as high COL1A1 levels and ECM turnover in response to increased IL-1 (89).

In labral cells, IL-1 triggers ECM degradation *via* secretion of IL-6, MMP-1/-2/-3/-9/-13, and ADAMTS-4/-5, while suppressing COL1A1 and COL2A1 expression. The secreted MMPs, ADAMTSs, and IL-6 are known to increase systemic cartilage damage and joint inflammation. Labral cells are unique in their increased MMP-9 expression after exposure to IL-1; MMP-9 is an inducible gelatinase which specifically degrades type IV and V collagen (89).

## Summary of Appraised Information: Factors Related to Relaxin and Hip Injuries

One aim of this scoping review is to show that it is scientifically logical that a correlation would exist between relaxin levels and the high incidence of soft tissue hip injuries in women. This review appraised available information on factors related to the issue of interest. To derive an abridged synopsis of the topics covered, it is helpful to revisit the comparatively large pool of literature that is available which addresses relaxin vs. ACL tears at the micro and macro level.

Beginning at the macro level, numerous studies correlate increased SRC with increased incidence of ACL tear. Conversely, decreasing SRC with OCPs decreased incidence of ACL tears. The impact of relaxin on the ACL during the menstrual cycle is facilitated by preceding estrogen and progesterone peaks which prime target tissues. Relaxin impacts only female ACLs, which display relaxin receptors with specific and saturable binding. Conversely, male ACLs display no relaxin receptors. This binding increases the susceptibility of the ACL to macro-physiologic injury, because micro-physiologic collagen degradation is upregulated while *de novo* synthesis is suppressed.

Beginning at the micro level for relaxin vs. hip soft tissue injuries, it is known that pelvic ligaments normally have a uniform distribution of relaxin receptors, and the receptor and hormone levels significantly increase in pathologic states such as uterine prolapse. It is also known that acetabular labral cells can be induced to express collagen-degrading MMPs while suppressing deposition of new collagen. The response is unique in the high expression levels of MMP-9; an inducible gelatinase crucial in the female reproductive system for endometrial remodeling during menstruation and basement membrane degradation capacity. MMP-9 is also well-known among pulmonologist for destroying a serine protease inhibitor, facilitating tissue breakdown at inflammatory sites.

The labral cells are adjacent to the ovaries, where the recurring corpus luteum synthesizes large amounts of the paracrine hormone relaxin during the mid-luteal phase. Relaxin remodels endometrial tissue, acting *via* MMP-9, expression of which also peaks just after ovulation. Relaxin modulates tissue dissolution by stimulating collagenases (MMP-1/-13) and gelatinases (MMP-2/-9); MMPs expressed by labral cells, particularly MMP-9.

**Figure 3:**
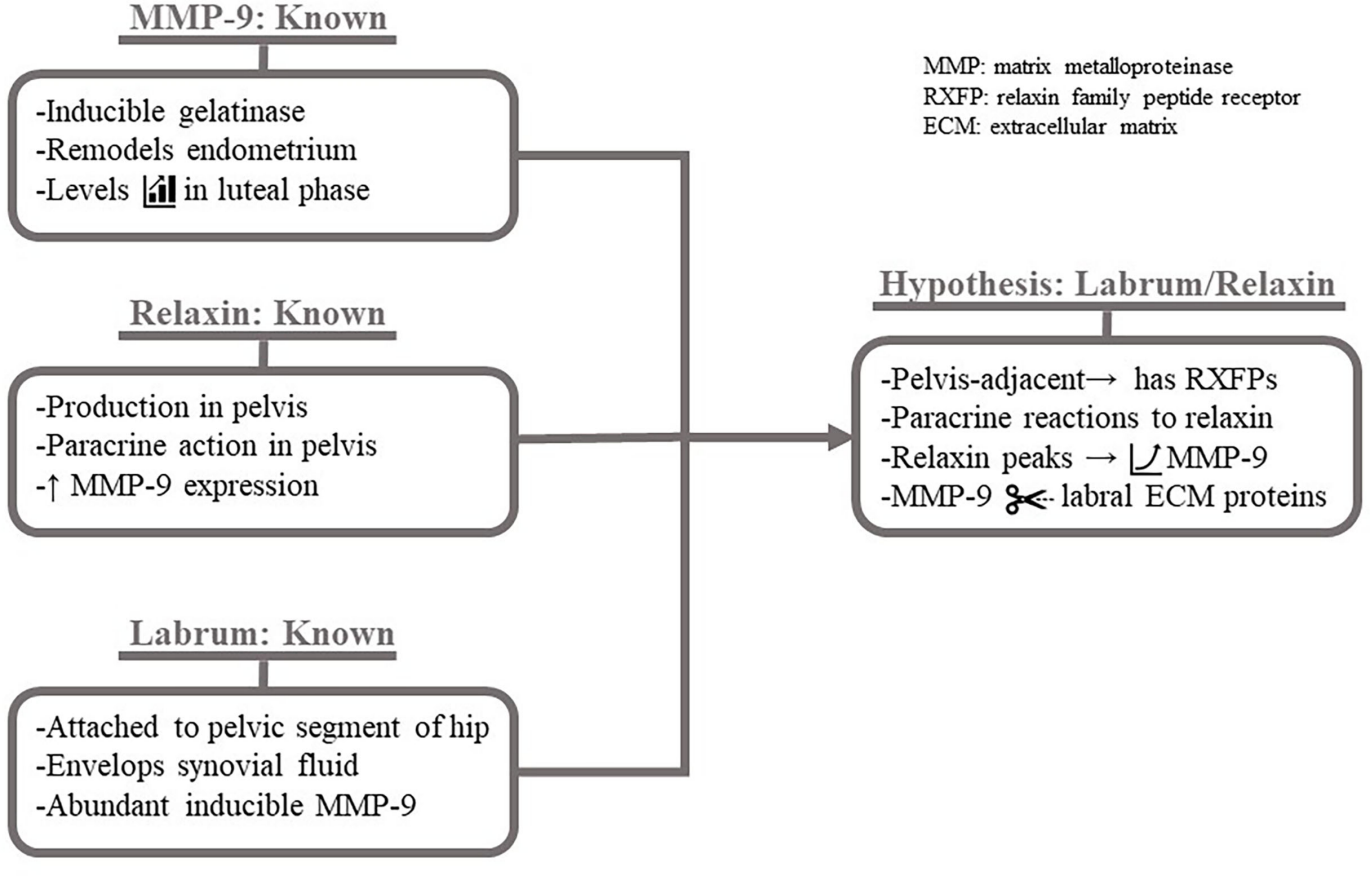
Hypothesized mechanism of relaxin-induced acetabular labrum damage. Known factors giving rise to hypothetical impact of relaxin on the acetabular labrum. It is known that MMP-9 is the inducible gelatinase (MMP-2 is not) which is expressed at high levels during the luteal phase for endometrial. It is known that relaxin, produced in the pelvis and exerting paracrine effects, increases MMP-9 expression. Finally, it is known that the anchored to the pelvis serves as a "seal" enclosing synovial fluid, which may contain hormones, and that the cells express MMP-9 at an unusually high level when induced . Given these known factors: the acetabular labrum likely expresses RXFPs and increases MMP-9 expression in a paracrine to relaxin. MMP-9 degrades ECM proteins, weakening the labrum.

Therefore, since relaxin acts in a paracrine fashion, it would be predicted to act throughout the female pelvis during the luteal phase. Due to the presence of relaxin receptors on uterosacral ligaments, ligament laxity would occur. If relaxin has receptors on the neighboring acetabular labral tissue, binding will also trigger collagen turnover *via* MMP-1/-2/-3/-9/-13 and other components. Labral cells have inducible high expression of MMP-9, which is upregulated in luteal phase reproductive tissue, implicating a large potential role for MMP-9 in relaxin-induced degradation of the acetabular labrum. The trio of known molecular components—MMP-9 properties, relaxin properties, and, recently, the metabolism of labral cells—hypothetically working in reciprocal and mutual ways to target the collagen of the acetabular labrum is detailed in **Figure 3**.

As seen in women with SPD, the micro level degradation of ligaments from high relaxin resulted in macro level instability of the pelvis. As hypothesized in recent DDH studies, relaxin-induced laxity of the pelvis vs. hips is interrelated: mothers with elevated relaxin causing pelvic instability are more likely to give birth to infants with DDH, who also display pelvic instability on early exams. In macro level research of the other ball-and-socket joint in humans—the glenohumeral joint—a significant correlation of increased relaxin levels with acute episodes of instability was found.

In addition to appraising available information on factors relevant to a potential hip injury/relaxin relationship, this scoping review also aimed to identify conceptual gaps for future research. The two main gaps in knowledge/research that were identified are as follows:

## Conceptual Gap 1: Do Soft Tissues of the Femoroacetabular Joint Respond to Relaxin?

There is, in essence, a “missing arrow” in the hypothetical cascade starting at cyclic relaxin peaks and ending at an increased risk of soft tissue injuries of the hip. It is known that relaxin is synthesized in the pelvis, has paracrine capabilities, and upregulates expression of the same collagen-degrading MMPs present on the acetabular labrum. What is not known, the “missing link”, is whether cells of the acetabular labrum, hip joint synovium, and other soft tissues of the hip joint contain the relaxin receptors necessary to bridge the route from relaxin peaks to increased risk of female hip injuries.

## Conceptual Gap 2: How Can Detrimental Musculoskeletal Side Effects of Relaxin be Countered?

It is known that relaxin is crucial for correct function/fertility of the female reproductive tract. Therefore, a theoretical broad “anti-relaxin” would prove harmful. However, it is also apparent that many musculoskeletal effects of relaxin are not physiologically necessary nor desirable, such as hip and knee injuries in female athletes. This presents the question of how to decrease detrimental side effects of relaxin peaks on the musculoskeletal system without causing iatrogenic issues. There are likely multiple avenues by which to approach this issue. Studies in the present review highlight problematic effects of relaxin, but also bring up potential mitigating factors, such as OCP use and menstrual cycle tracking.

## Limitations

Due to the veritable absence of any type of literature discussing women’s hip injuries in the context of relaxin, the premise of this review is based entirely on inductive reasoning. Additionally, consideration of lower-level of evidence literature and inconclusive literature was necessary.

## Summary of Scoping Review

The present review showed the scientific logic and medical importance of further researching the potential causative role of relaxin in female hip injuries. Appraisal of available information on related factors, and identification of conceptual gaps for future research was completed.

Collectively, the factors examined in this review support the inference that relaxin impacts the female predominance of soft tissue hip injuries. The steps from relaxin peak to increased ACL injury risk are likely highly analogous to the methodology by which relaxin can precipitate hip damage. Relaxin’s synthesis in the pelvis and paracrine profile of action support the potential of relaxin/hip interactions.

This review also highlights why relaxin’s sex-specific musculoskeletal effects should be a major orthopedic research focus. The first conceptual gap, bridged simply by assessing male vs. female hip tissue for relaxin receptors, could show a female-specific, predictable susceptibility to devastating lower extremity injuries. Subsequently, multiple considerations for the second conceptual gap, attenuating relaxin-associated musculoskeletal damage in women, would be critical to explore in order to provide quality, equitable orthopedic care to male and female patients.

## Conclusion

Menstrual cycle peaks of relaxin activate MMPs, which locally degrade collagen and gelatine. Women have relaxin receptors in multiple joints, and increased relaxin correlates with increased musculoskeletal injuries. Relaxin has paracrine effects in the female pelvis on ligaments adjacent to hip structures, such as acetabular labral cells and hip capsule/synovial cells which express high levels of relaxin-targeted MMPs. Therefore, it is imperative to investigate the effect of relaxin on the hip to determine if increased levels of relaxin are associated with an increased risk of acetabular labral tears.

## Data Availability Statement

The original contributions presented in the study are included in the article/**Supplementary Material**. Further inquiries can be directed to the corresponding author.

## Author Contributions

Per the International Committee of Medical Journal Editors Recommendations for the Conduct, Reporting, Editing, and Publication of Scholarly Work in Medical Journals (ICMJE) Recommendations 2019. EP—author, per ICMJE 2019 recommendations. AM—author, per ICMJE 2019 recommendations. JG—author, per ICMJE 2019 recommendations. MW—author, per ICMJE 2019 recommendations. RW—author, per ICMJE 2019 recommendations. JB—contributor, per ICMJE 2019 recommendations. EP conceived the original review idea, and presented this idea to AM, JG, MW, and RW to direct further topic exploration and refinement of the review focus. JB advised the authors on the design of the work, providing scoping review information. EP ran preliminary PubMed literature searches before EP, AM, and JB met to tailor the search strings for data acquisition. EP and AM performed preliminary data analysis *via* the paper screening process, with oversight and input from JG, MW, and RW with regard to screening directions, such as inclusion or exclusion of animal-only subjects. EP and AM then performed more detailed analysis of included studies and interpretation of the charting results, with guidance from JG, MW, and RW on matters such as appropriate categories into which data could be grouped and assessed (i.e. the musculoskeletal effects of relaxin category). EP and AM drafted the manuscript, with significant changes to manuscript organization implemented by JG, MW, and RW. All authors then continued to revise the manuscript until, by consensus, each gave the current version final approval for publication. All authors agree to be held accountable for accuracy and integrity of the work.

## Conflict of Interest

All authors have completed the ICMJE uniform disclosure form at www.icmje.org/coi_disclosure.pdf and declare as follows: MW reports personal fees from Depuy Synthes Sales Inc, other from Smith & Nephew, personal fees from Zimmer Biomet Inc, personal fees from Stryker Corp; outside the submitted work. RW reports personal fees, non-financial support and other from Smith and Nephew, other from Arthrex, personal fees from Medical Device Business Systems, personal fees from Linvatec Corporation, other from Wardlow Enterprises; outside the submitted work.

The remaining authors declare that the research was conducted in the absence of any commercial or financial relationships that could be construed as a potential conflict of interest.

## Publisher’s Note

All claims expressed in this article are solely those of the authors and do not necessarily represent those of their affiliated organizations, or those of the publisher, the editors and the reviewers. Any product that may be evaluated in this article, or claim that may be made by its manufacturer, is not guaranteed or endorsed by the publisher.
