# Supplementary material for: Do Relaxin Levels Impact Hip Injury Incidence in Women? A Scoping Review
Source: Front Endocrinol (Lausanne). 2022 Feb 4;13:827512. doi: 10.3389/fendo.2022.827512 (PMC8855110; doi:10.3389/fendo.2022.827512)
Supplement: Supplementary file 1 [file DataSheet_1.zip › Appendix 3.DOCX]

| **Appendix 3: All Included Studies in Present Review** | | | | |
| --- | --- | --- | --- | --- |
| **Cellular/Molecular Effects of Relaxin** | | | | |
| **Author, Year** | **Type of Study** | **Patients (% F)** | **Study Intervention or Condition of Interest** | **Study Conclusions Relevant to Relaxin and/or General Study Conclusions** |
| Ando et al, 1960^23^ | Controlled Laboratory | - | Pelvic relaxation after relaxin in guinea pigs | Increased levels of administered relaxin increased the number of guinea pigs that had pelvic relaxation greater than 0.3 mm but did not increase the distance of relaxation. |
| Braddon, 1982^29^ | Prospective Cohort Animal | - | Effect of Relaxin on cAMP^‡^ levels in the pubic symphysis | Relaxin increases cAMP^‡^ in target tissue and ornithine decarboxylase produces relaxin. |
| Bryant-Greewood et al, 1982^30^ | Review | - | Effects of relaxin on fibroblasts, uterus, and cervix | Relaxin is associated with increased hydroxyproline incorporation in collagen and bind in the uterus, cervix, and uterus. |
| Dragoo et al, 2003^10^ | Controlled Laboratory | 10 (50%) | Relaxin receptors on human ACLs^†^ | Relaxin exhibits specific saturable binding in the female anterior cruciate ligament, where specific relaxin receptors were present. |
| Ferlin et al, 2010^36^ | Controlled Laboratory | - | Relaxin’s effect on osteoclasts | Relaxin induces differentiation and activation of human osteoclasts. |
| Galey et al, 2003^38^ | Controlled Laboratory | 12 (67%) | Relaxin staining of ACL^†^ samples | ACL^†^ samples stain positively for relaxin indicating the presence of relaxin receptors. |
| Goldsmith et al, 1995^40^ | Review | - | Relaxin and pregnancy | Relaxin is associated with pelvic or pubic-symphyseal relaxation. |
| Grossman et al, 2010^42^ | Review | - | Relaxin’s effect on acute heart failure | Relaxin plays a role during pregnancy and also is a vasodilator in male and nonpregnant females which could be a potential treatment for acute heart failure. |
| Henneman et al, 2008^16^ | Controlled Laboratory | - | Effect of relaxin on gelatinase expression of human periodontal ligament cells | Dose dependent increase of MMP^#^-2 production and alpha-smooth muscle actinin expression. Total MMP^#^ activity was not affected. |
| Kapila, Xie 1998^47^ | Prospective Cohort Animal | - | Modulation of MMPs^#^ and proteinase inhibitors by relaxin | Relaxin produced a dose-dependent induction of collagenase-1 and stromelysin-1, but minimal modulation of TIMP-1^***^ and TIMP-2^***^ expression in the fibrocartilaginous cells. |
| Kapila et al, 2013^46^ | Controlled Laboratory | - | Relaxin receptors effect on induction of MMPs^#^ in TMJ^†††^fibrochondrocytes. | Relaxin and estrogen enhance matric loss in the TMJ^†††^disc by inducing MMP^#^-9 and MMP^#^-13. |
| Kleine et al, 2017^49^ | Review | - | Relaxin’s role on ligaments and joints | Relaxin is important in modulating ligament laxity during pregnancy and has been recently implicated as a regulator of collagen metabolism. It has been detected in the synovial lining of patients undergoing arthroplasty. |
| Lubahn et al, 2006^53^ | Prospective cohort | 8 (100%) | Presence of relaxin receptors in volar oblique ligament | Relaxin binds to the volar oblique ligament with specificity, implying a receptor-mediated process. |
| MACL^†^ennan et al, 1983^54^ | Review | - | Effects of relaxin on reproductive system | Relaxin facilitates the remodeling of connective tissue in target tissue during pregnancy and parturition. |
| Nistri et al, 2007^60^ | Review | - | Biological effects of relaxin | Relaxin is a potent vasodilator of the systemic and coronary circulation. |
| Nose-Ogura et al, 2017^13^ | Prospective Cohort | 106 (100%) | Serum relaxin levels during phases of menstrual cycle, OCP^**^ effect on relaxin levels | Relaxin levels were increased during luteal phase of menstrual cycle. OCP^**^ decreased relaxin concentration in participants with high relaxin levels. |
| Powell et al, 2015^67^ | Review | - | Effect of relaxin on MMP^#^ production and activity | Relaxin alter tissue degradation and repair by modulating MMPs^#^. |
| Ren et al, 2015^69^ | Controlled Laboratory | - | R2 effect on cell migration, invasiveness, and proliferation of osteosarcoma cells. | R2 increases cell migration, invasiveness, and proliferation of osteosarcoma cells possibly through calcium-binding proteins/MMP^#^-9 signaling. |
| Samuel et al, 2004^73^ | Prospective Cohort Animal | - | Relaxin & relaxin receptors in cardiac tissue, effects on cardiac fibrosis | Relaxin inhibits cardiac collagen secretion and deposition by directly inhibiting cardiac fibroblast proliferation, differentiation, and activation and increasing MMP^#^ activity. |
| Steinetz et al, 1967^78^ | Controlled Laboratory | - | Effects of relaxin, estrogen, and progesterone of mouse pelvises | Combination of estrogen and relaxin produce the greatest amount of interpubic ligament lengthening |
| Van der Meer, 1950^82^ | Prospective Cohort Animal | - | Symphysis pubis stretching in the presence of relaxin | The symphysis pubis has greater relaxation when treated with estrogen and relaxin but less of a response under anesthesia or spinal cord sectioned. |
| Veitia et al, 1998^83^ | Controlled Laboratory | - | Relaxin gene location | 2 relaxin genes (RLN1, RLN2) and insulin gene INSL-4 are all on chromosome 9 and clustered in the same region. |
| Wolf et al, 2013^86^ | Controlled Laboratory | 992 (16%) | Serum relaxin levels | Men and women had detectable serum relaxin levels and did not correlate with generalized joint laxity. |
| Wolf et al, 2012^17^ | Controlled Laboratory | 49 (61%) | Serum relaxin levels, and activity of MMP^#^-1 and -3 | Relaxin receptors in the anterior oblique ligament and MMP^#^-1 and -3 are upregulated by relaxin binding. |

| **Systemic-Musculoskeletal Effects of Relaxin** | | | | | | | | |
| --- | --- | --- | --- | --- | --- | --- | --- | --- |
| **Author, Year** | **Type of Study** | | **Patients (% F)** | | **Study Intervention or Condition of Interest** | | **Study Conclusions Relevant to Relaxin and/or General Study Conclusions** | |
| Alves de Oliveria et al, 2012^12^ | Prospective Case Control | | 52 (100%) | | Oral contraceptive effect on chewing amplitudes in women with and without disc displacement | | Hormonal fluctuation from oral contraceptives does not influence the jaw amplitudes or opening and closing velocity in patients with or without disc displacement | |
| Arnold et al, 2002^8^ | Prospective Case Control | | 62 (92%) | | Serum relaxin levels and joint translation in ACL^†^ injured, non-injured, and non-athletes | | Relaxin fluctuates weekly, females with prior ACL^†^ injury have greater joint laxity, no change in laxity over menstrual cycle, laxity does not correlate with relaxin levels. | |
| Brophy et al, 2010^7^ | Review | | - | | Risk factors and prevention for ACL^†^ injuries | | The menstrual cycle is complex and there is little known about the effect relaxin, estrogen, and testosterone have on the ACL^†^. | |
| Charalambous, et al, 2012^32^ | Review | | - | | Female sex hormone effect on myofibroblasts and post-traumatic arthritis | | Relaxin may act on extracellular matrix and myofibroblasts influencing joint laxity and fibrosis. Increased laxity is more common in females and during pregnancy. There are estrogen, progesterone, and relaxin receptors in the ACL^†^ with increased laxity during estrogen and progesterone peaks during menstrual cycle. | |
| Clifton et al, 2012^33^ | Prospective Cohort | | 6 (67%) | | Immunostaining for relaxin receptor in dorsoradial ligaments and synovium | | Females has a greater amount and intensity of immunostaining for relaxin receptors. | |
| DeFroda, et al, 2019^11^ | Retrospective Cohort | | 165,748 (100%) | | Effect of OCPs^**^ on ACL^†^ reconstruction rates | | OCPs^**^ have a protective effect on ACL^†^ tears especially in the 15-19 age group with a 63% reduction in the tear rate. | |
| Deniz et al, 2012^14^ | Prospective Case Control | | 50 (100%) | | Relaxin serum levels between women with TMJ^†††^internal derangement and those without | | Patients with TMJ^†††^internal derangements have higher serum relaxin levels than those without which may explain the increased prevalence of TMJ^†††^internal derangement in women. | |
| Deniz et al, 2013^34^ | Prospective Case Control | | 28 (93%) | | Serum relaxin levels in patients with TMJD^†††^ and the association with osteoarthritis and/or joint effusion | | Increased levels of relaxin may influence the presence of joint effusion but not osteoarthritis in patients with TMJ^†††^disorder. | |
| Dragoo, et al, 2011^1^ | Prospective Cohort | | 128 (100%) | | Serum relaxin concentration association with ACL^†^ tears during collegiate athletics career | | Elite female athletes with ACL^†^ tears have higher serum relaxin levels than those without tears. Serum relaxin >6.0pg/mL had more than 4 times increased risk of ACL^†^ tear. | |
| Dragoo, et al, 2011^9^ | Cross-Sectional | | 169 (100%) | | Serum relaxin and progesterone levels compared between female college athletes taking OCPs^**^ and those not | | Positive correlation between serum progesterone and serum relaxin concentration and a decrease of serum relaxin concentration with hormonal contraceptive use. | |
| Em et al, 2015^35^ | Prospective Case Control | | 88 (100%) | | Serum relaxin levels in patients with benign joint hypermobility syndrome and those without | | Serum relaxin levels are significantly elevated in patients with benign joint hypermobility syndrome; including arthralgia, shoulder impingement syndrome, pes planus, and hyperkyphosis. | |
| Gates, et al, 2005^39^ | Review | | - | | Relaxin’s antifibrotic effects after musculoskeletal injury | | Relaxin reduces type I and III collagen production and increased the action of collagenases. | |
| Gould, et al, 2016^41^ | Review | | - | | Risk factors for ACL^†^ injury in females | | It is difficulty to assess the precise effect of circulating estrogen and relaxin on the mechanical properties of the ACL^†^. | |
| Huston, et al, 2000^45^ | Review | | - | | Risk factors for ACL^†^ injuries in females | | 2 studies found conflicting results concerning peak incidence of ACL^†^ injuries when compared to the menstrual cycle. Women on OCPs^**^ had less traumatic injuries while playing handball. | |
| Komatsu, et al, 2018^50^ | Review | | - | | Anatomy and hormonal response of the carpometacarpal joint | | Relaxin upregulates MMPs^#^ and suppresses tissue inhibitors or MMPs^#^. The anterior oblique ligament binds relaxin causing attenuation of the ligaments or inhibition of repair especially during reproductive years. | |
| McGorray, et al, 2012^59^ | Randomized Control Trial | | 39 (72%) | | Tooth movement after weekly 50 ug injections of relaxin | | No differences in tooth movement during 8 weeks of injections of 4 weeks after treatment. | |
| Noon, et al, 2012^61^ | Review | | - | | The effects of hormones on low back pain in pregnant athletes | | Estrogen increases the relaxin receptor sensitivity. Patient with most severe back pain had higher levels of relaxin. | |
| Owens, et al, 2016^62^ | Prospective Cohort | | 106 (11%) | | Comparing the relaxin levels in patients with shoulder instability to those without | | Mean serum relaxin concentration was higher in the shoulder instability group. The risk of shoulder instability more than doubled during 4-year follow up with every 1 pg/mL increase in baseline relaxin concentration. | |
| Pearson, et al, 2011^64^ | Prospective Cohort | | 12 (100%) | | Relaxin concentration and tendon laxity over the course of the menstrual cycle. | | Higher relaxin concentrations was associated with decreased patellar tendon stiffness. | |
| Pokorny, et al, 2000^66^ | Prospective Cohort | | 55 (100%) | | Laxity of knee and hand between oral contraceptive users and non-users | | No differences in hand or knee laxity in OCP^**^ users compared to non-users. | |
| Szalay, 2012^79^ | Review | | - | | Musculoskeletal differences between men and women | | Research is ongoing to elucidate the basis of both physiological dimorphism and gender disparity, but in most instances, causative factors remain unclear. | |
| Toth, et al, 2001^80^ | Review | | - | | ACL^†^ injuries in female athletes | | Estrogen and relaxin level fluctuations could contribute to decreased knee stability in females. Contradicting studies on if OCPs^**^ are protective or not. High levels of relaxin can increase risk of ligament injuries during pregnancy. | |
| Warren, et al, 2001^84^ | Review | | - | | TMJ^†††^disorders are possible explanations for women predominance | | Relaxin may contribute to aberrant TMJ^†††^remodeling. | |
| Wolf, et al, 2013^4^ | Prospective Cohort | | 289 (53%) | | Serum relaxin levels, effect on joint laxity | | Increased relaxin levels were associated with increased trapezial-metacarpal joint laxity but not generalized joint laxity. | |
| Wolf, et al, 2011^15^ | Prospective Cohort | | 49 (61%) | | Serum relaxin levels and relaxin receptor concentration | | Higher level of serum relaxin is correlated with increased numbers of relaxin receptors in the thumb ligaments and has an impact on upregulation of MMP^#^-1. | |
| **Pelvic Related Effects of Relaxin** | | | | | | | | |
| **Author, Year** | | **Type of Study** | | **Patients (% F)** | | **Study Intervention or Condition of Interest** | | **Study Conclusions Relevant to Relaxin and/or General Study Conclusions** |
| Bookhout et al, 1996^26^ | | Review | | - | | Pelvic musculoskeletal dysfunction per hormonal events of female life cycle | | Women’s SI^¶¶^  joints have smaller, less congruent articulating surfaces. Combined with the ligamentous laxity due to relaxin peaks throughout the female lifespan, women are left more susceptible to pelvic musculoskeletal pathologies. |
| Borg-Stein et al, 2007^27^ | | Review | | - | | Identification and management of pre-, intra-, and post-partum musculoskeletal disorders | | Relaxin remodels pelvic tissue and activates collagen degrading pathways, and is present in high levels during pregnancy. This may be responsible for the pelvic and SI^¶¶^  pain common in pregnancy, |
| Camiel et al, 1986^31^ | | Retrospective Cohort | | 100 (100%) | | The relaxin-mediated pubic sympyseal fissure during pregnancy | | During pregnancy, relaxin loosens pelvic ligaments, allowing the PS^‡‡^ to widen and have increased flexibility. The widening can be visualized as a vertical fissure on x-ray. |
| Kieserman-Shmokler et al, 2020^48^ | | Review | | - | | Relaxin interaction with apical ligaments, role in prolapse | | The uterosacral ligaments of women with pelvic organ prolapse had higher levels of flexible type III collagen, higher relaxin levels, increased MMP^#^-1/-9 expression, and more loosely packed collagen fibers. |
| Kristiansson et al, 1996^51^ | | Prospective Cohort | | 200 (100%) | | Relationship of serum relaxin vs. back pain during pregnancy | | Mean SRC^##^ during pregnancy positively correlated with PS^‡‡^ or low back pain during late pregnancy. Relaxin levels returned to undetectable levels until 3 months post-partum. |
| Leadbetter et al, 2004^52^ | | Review | | - | | Management of symphysis pubis dysfunction in pre- and post-partum women | | The weakening of pelvic joints during pregnancy is thought to be due to relaxin, with higher SRC^##^ in gravid women with pelvic pain and the highest SRC^##^ levels in gravid women incapacitated by pelvic/SI^¶¶^  pain. SRC^##^ returns to normal at 4-12 weeks postpartum. Increased relaxin has also been associated with pelvic and low back pain during menstruation. |
| MACL^†^ennan et al, 1991^55^ | | Review | | - | | Association of high serum relaxin and symptomatic pelvic girdle relaxation during pregnancy | | Relaxin, produced by the corpus luteum of pregnancy, induces pelvic relaxation via paracrine action. In some women the degree of pelvic girdle relaxation is greater than normal/greater than necessary. These women are more likely to have pelvic pain and instability during pregnancy. This may be due to increased levels of relaxin, and/or increased susceptibility to the hormone. |
| Mac Lennan et al, 1986^56^ | | Case-Control | | 35 case (100%), 368 control (100%) | | Relaxin immunoreactivity in pregnant women with severe pelvic joint pain and instability | | The serum relaxin immunoreactivity of women with severe pelvic pain and instability during late pregnancy was significantly higher compared to asymptomatic women during late pregnancy; often above the 95% CI of the median among the corresponding gestational age in the control group. Patients with the highest relaxin levels were the most functionally incapacitated by their symptoms. |
| Owens et al, 2002^63^ | | Review | | - | | PS^‡‡^ separation during pregnancy and symphysis pubis disruption | | Relaxin is thought to allow pelvic laxity for parturition, as non-pregnant studies have shown 2600lbs of force is normally required to separate the PS^‡‡^ joint. However, excess relaxin has been associated with disabling pelvic girdle pain persisting long after birth. |
| Pires et al, 2015^65^ | | Case Report and Review | | 1 (100%) | | Relaxation of the PS^‡‡^ and SI^¶¶^  joints during pregnancy | | Relaxin and progesterone facilitate birth canal widening by relaxing pelvic ligaments. However, this can cause functional pelvic pain, and in rare cases, acute symphyseal disruption. |
| Reisenauer et al, 2010^68^ | | Conference; Controlled Laboratory | | 39 (100%) | | Smooth muscle regulation in the sacrouterine ligament | | In vitro, the smooth muscle components of the uterosacral ligaments are regulated by sympathetic/parasympathetic neurotransmitters, which are modulated by relaxin and oxytocin. |
| Ritchie et al, 2003^71^ | | Review | | - | | Common orthopedic conditions occurring during pregnancy | | Pregnant women with the highest SRC^##^s have the most debilitating levels of low back pain; relaxin has also been identified as a significant contributor to pubic symphysis pain. |
| Saugstad et al, 1991^75^ | | Cross-Sectional | | 153 (100%) | | Persistent post-partum pelvic pain and pelvic joint instability (PPPJI^††^) | | Pregnant women impacted by severe/incapacitating PPPJI^††^ have significantly higher SRC^##^. Symptom onset was earlier in women with prior OCP^**^ use. Effected women were more likely to have post-term deliveries, infants with higher birth weights, female infants, and infants with congenital hip dysplasia. |
| Schott et al, 2014^76^ | | Controlled Laboratory | | 43 (100%) | | Presence of relaxin and oxytocin in the pelvic ligaments of prolapse patients | | Levels of relaxin-2 were significantly higher in the uterosacral ligaments of pelvic organ prolapse patients, compared to controls. |
| Schuster et al, 1977^21^ | | Review | | - | | Hormonal etiology of abnormal pronation in children | | A potential cause of DDH^¶^ pathogenesis being predominant in females is that effected infants are hypersensitive to estrogen and relaxin; possibly an inborn genetic error of estrogen metabolism |
| Weinberg et al, 1956^85^ | | Case-Control | | 15 (100%) | | PS^‡‡^ separation during pregnancy | | In pregnant and nonpregnant women, relaxin extract administered via injection for 1-5 days (total dosage 100mg) did not change radiographic pelvic measurements. |

| **Hip Related Effects of Relaxin** | | | | |
| --- | --- | --- | --- | --- |
| **Author, Year** | **Type of Study** | **Patients (% F)** | **Study Intervention or Condition of Interest** | **Study Conclusions Relevant to Relaxin and/or General Study Conclusions** |
| Andren et al, 1962^18^ | Book Chapter | 64 cases (80%), 349 controls (53%) | Concurrent pelvic instability and DDH^¶^ in infants; hormonal factors | Instability of the pelvis in neonates with hip dislocation is similar in character to the pelvic instability experiences by pregnant women, attributable to relaxin. Neonatal hip dislocation may reflect increased number of relaxin receptors, or increased reactivity of the receptors present. Androgenic hormone antagonism of estrogen and relaxin decreases the likelihood of DDH^¶^ in males. Although neonatal hip dislocations often spontaneously reduce within days after birth, unless detected and treated, recurrent subluxation will almost always develop at a later date. |
| Bracken et al, 2012^28^ | Review | - | DDH^¶^ risk factors, diagnostic controversies, and current management options | DDH^¶^ is more common in women with symptomatic pelvic girdle relaxation, possibly due to increased susceptibility to relaxin among mother and infant. The collagenolytic enzymes triggered by relaxin increase cell flexibility. However, cord blood relaxin levels and infant SRC^##^ do not correlate with incidence of DDH^¶^. |
| Forst et al, 1997^37^ | Cross-Sectional | 90 (55%) | Correlation of relaxin levels and neonatal hip joint stability | Ultrasound showed increasing neonatal hip instability correlating with decreased cord blood relaxin concentration. This may reflect low relaxin levels yielding insufficient preparation of the birth canal. |
| MACL^†^ennan et al, 1997^19^ | Survey-Based | 1115 (100%) | The association of symptomatic pelvic girdle relaxation during pregnancy with postnatal pelvic joint pain, and neonatal DDH^¶^ | Pelvic pain in pregnancy began with the first pregnancy in 74% of respondents, worsening with subsequent pregnancies and persisting for more than 6 years post-partum. The PS^‡‡^ and SI^¶¶^  joint were most commonly impacted, but peripheral joint pain occurred as well. Many of these women had strong family histories of maternal pelvic joint syndrome and neonatal DDH^¶^. The DDH^¶^ incidence among infants born to these mothers was 45/1000; 5x the average incidence for the country. |
| Morey et al, 2001^20^ | Practice Guideline Article | - | American Association of Pediatrics guidelines for early DDH^¶^ detection | When assessing contributing factors: DDH^¶^ is more common in female infants, likely due to increased susceptibility to maternal hormone relaxin. In unilateral cases, the left hip is impacted 3x more often than the right, likely due to typical intrauterine positioning of infants. |
| Rhodes et al, 2014^70^ | Review | - | Environmental factors impacting DDH^¶^ | DDH^¶^ predisposing factors may be congenital or manifest later; they can be prenatal or postnatal. Previously identified risk factors include positive family history, firstborn status, breech birth, and female gender; newer research is examining the potential role of relaxin, vitamin C, and vitamin D. |
| Roof et al, 2013^72^ | Review | - | Screening options and techniques for DDH^¶^ | DDH^¶^ etiology is important to the evaluation process. The hips of female infants are more sensitive to relaxin-induced ligamentous laxity, thus increased maternal relaxin may contribute to higher DDH^¶^ incidence in female infants. |
| Schuster et al, 1977^21^ | Review | - | Potential hormonal etiology of abnormal pronation in children | Relaxin is postulated to significantly impact DDH^¶^ pathogenesis, with estrogen and progesterone. These hormones and their pathophysiological effects may also impact ligaments of the foot, leading to abnormal pronation in children. |
| Uden et al, 1988^81^ | Retrospectve Cohort | 11 (72.7%) | Incidence of inguinal hernia in infants with DDH^¶^ | Female infants with DDH^¶^ were 4.8x more likely to require inguinal hernia repair during the first year of life; male infants with DDH^¶^ had a 2.8s increased likelihood. Female infants may have an increased association because they are exposed to increased levels and/or have increased sensitivity to maternal relaxin, a collagenase stimulant. |

| **Potential Relaxin Benefits, Mitigatory Options^*^** | | | | |
| --- | --- | --- | --- | --- |
| **Author, Year** | **Type of Study** | **Patients (% F)** | **Study Intervention or Condition of Interest** | **Study Conclusions Relevant to Relaxin and/or General Study Conclusions** |
| Armfield et al, 2006^24^ | Review | - | Lower extremity muscle injuries commonly seen in athletes | Relaxin utilized as a growth factor via gene therapy can actually assist with healing of skeletal muscle injury. |
| Grossman et al, 2010^42^ | Review | - | Relaxin as a treatment for congestive heart failure | After its initial discovery and research in the context of pregnancy, relaxin receptors have since been found in a wide variety of organs, in men and women. Activated receptors act through multiple pathways, one of which activates endothelin type B and then nitric oxide. The resultant vasodilation increases cardiac output and renal perfusion. |
| Hamilton et al, 2012^43^ | Review | - | Orthopedic considerations in pregnant women, such as common musculoskeletal conditions and management of orthopedic trauma | Most of the common and potentially disabling musculoskeletal issues secondary to pregnancy are instigated by general soft tissue swelling and estrogen/relaxin-induced ligamentous laxity. Common complaints include hip pain, low back pain, pelvic pain, knee pain, and leg cramps. |
| Heckman et al, 1994^44^ | Review | - | Potential musculoskeletal issues of pregnancy | Relaxin has been associated with progression of scoliosis and the risk of pubic symphysis rupture during pregnancy. Relaxin levels are highest in the first trimester, decreasing to a stable level through parturition. |
| Marshall-Gradisnik et al, 2004^57^ | Conference; Prospective Cohort | 10 (100%) | Effect of monophasic OCP^**^ on relaxin, tumor necrosis factor (TNF)-alpha, and macrophage migration inhibitory factor (MIF) | Blood samples on CD^§^ 2, 16, and 26 taken from women on OCPs^**^ showed no significant changes in relaxin, TNF, or MIF. Specifically there was no peak of relaxin and other cytokines on CD^§^16, which is theorized to be the day with highest ACL^†^ injury risk. This suggests monophasic OCPs^**^ may help to reduce ACL^†^ injury risk by regulating relaxin levels. |
| Martin et al, 2019^58^ | Review | - | Beneficial cardiovascular effects of relaxin in pathological states | Relaxin is capable of suppressing arrhythmias, downregulating inflammation, and reversing fibrosis in patients with hypertension, atrial fibrillation, heart failure, or prior myocardial infarction. |
| Sanders et al, 2017^74^ | Longitudinal Community Health | 10,153 | Genome-wide association study of TMJD^†††^ in the US Hispanic Community | In sex-stratified analysis, a locus abnormally replicated among female TMJD^†††^ patients was immediately upstream from RXFP2^§§^ coding. |
| Snith et al, 2002^77^ | Review | - | Musculoskeletal differences and injury risk in male, female athletes | In women, relaxin induces ligament and tendon laxity, causing female athletes to be susceptible to a different set of injuries compared to male athletes. |
| Wolf et al, 2009^5^ | Review | - | Factoring relaxin-induced female hand/wrist ligamentous laxity into hand surgery care plans. | Relaxin receptors have been identified on soft tissues supporting the thumb CMC^*^ joint, such as the anterior oblique ligament. If relaxin-induced laxity causes the female predisposition for development of thumb CMC^*^ arthritis, treatments could be explored which locally reduce the impact of relaxin. |

**Footnotes Appendix Figure 3 (Alphabetical Order)**

^*^1st CMC: first/thumb carpometacarpal joint

^†^ACL(R): anterior cruciate ligament (repair)

^‡^cAMP: cyclic adenosine monophosphate

^§^CD: [menstrual] cycle day

^¶^DDH: developmental dysplasia of the hip

^#^MMP: matrix metalloproteinase

^**^OCP/HCP: oral/hormonal contraceptive

^††^PPPJI: pelvic pain and pelvic joint instability

^‡‡^PS: pubic symphysis

^§§^RXFP1 or 2: relaxin family peptide receptor 1 or 2

^¶¶^SI/SIJ: sacroiliac/sacroiliac joint

^##^SRC: serum relaxin concentration

^***^TIMP-1,-2: tissue inhibitors of MMPs

^†††^TMJ(D): temporomandibular joint (disorder)
