# Supplementary figures and images for: Do Relaxin Levels Impact Hip Injury Incidence in Women? A Scoping Review
Source: Front Endocrinol (Lausanne). 2022 Feb 4;13:827512. doi: 10.3389/fendo.2022.827512 (PMC8855110; doi:10.3389/fendo.2022.827512)

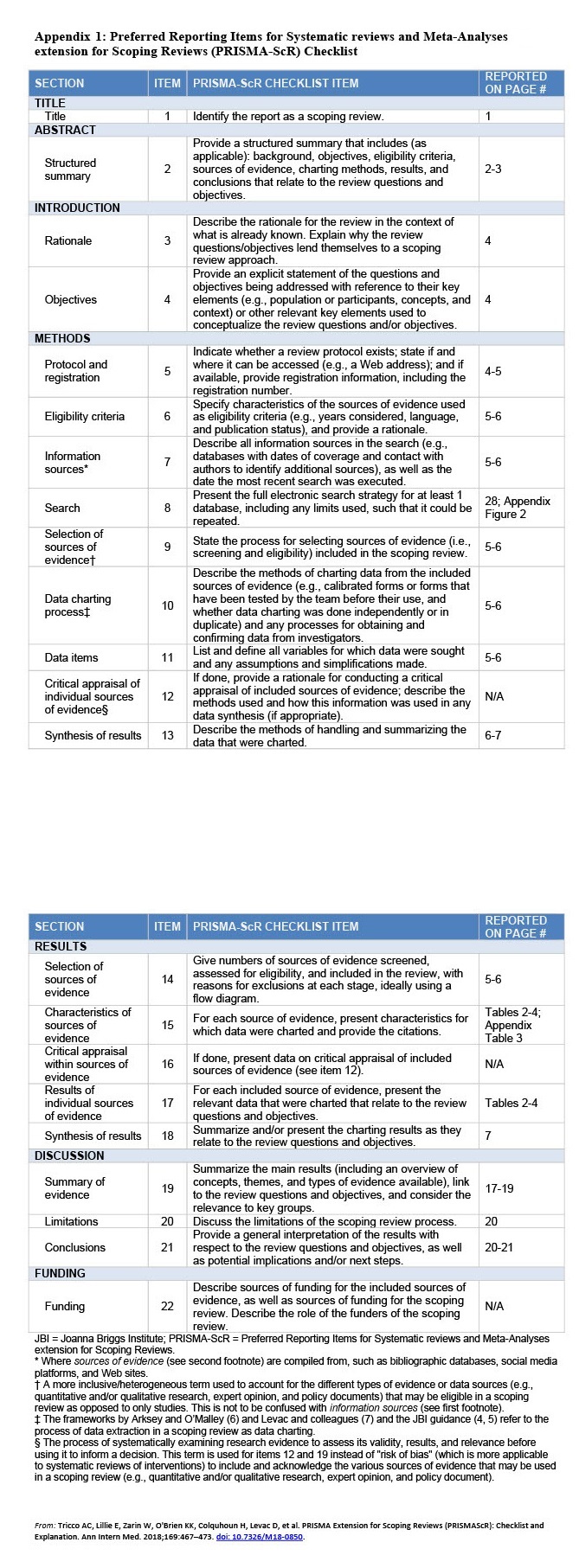

Supplement: Supplementary file 1 [file DataSheet_1.zip › Appendix 1.JPEG]

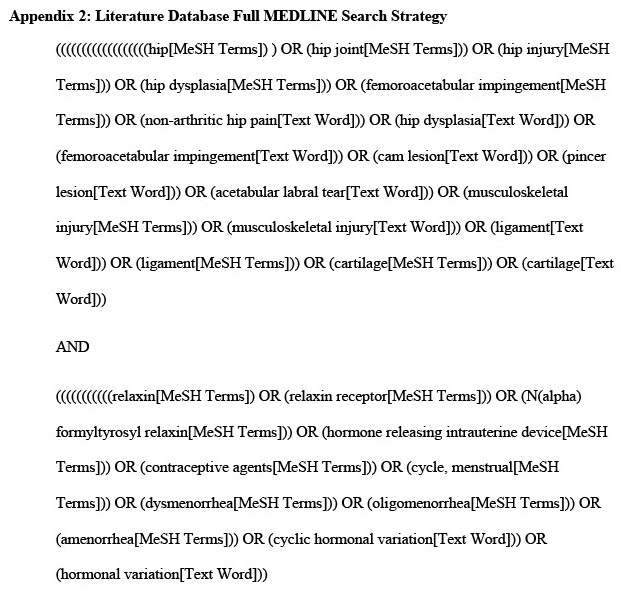

Supplement: Supplementary file 1 [file DataSheet_1.zip › Appendix 2.JPEG]
